# HLA association with HTLV-1/2 infection in different populations of Argentina

**DOI:** 10.1186/1742-4690-8-S1-A107

**Published:** 2011-06-06

**Authors:** Carolina Berini, Maria E Eirin, Richard Malan, Rogelio Espejo, Cecilia Delfino, Graciela Theiler, Mirna Biglione

**Affiliations:** 1 Centro Nacional de Referencia para el SIDA, Departamento de Microbiología, Facultad de Medicina, Universidad de Buenos Aires, Buenos Aires, Argentina; 2 Banco de Sangre Central de la Provincia de Misiones, Misiones, Argentina; 3 Hospital Rawson, Servicio Laboratorio Central, Sector Biología Molecular, San Juan, Argentina; 4 Laboratorio de Inmunogenética, Hospital de Clínicas, Buenos Aires, Argentina

## Introduction

HLA class 1 alleles HLA-A*24,*26 and HLA-B*07,*61 were associated to susceptibility for HTLV-1 infection.While HLA-A*02 was associated to protection to HAM/TSP, HLA-B*07 was associated to susceptibility for disease. Alleles HLA-A*02 and HLA-B*27,*40,*48 were described in aboriginal populations of Russia genetically related to Andean aborigines. This study analyzed the association of HLA with susceptibility to HTLV-1 or -2 infections.

## Materials and methods

60 negative samples from aboriginal populations, 23 HTLV-1 samples (12 from blood donors (BD), 11 from Kollas) and 32 HTLV-2 positive samples from Buenos Aires residents were analyzed. HTLV-2 positive individuals and BD were predominantly of Caucasian origin. HLA class 1 (A, B) characterization was performed on genomic DNA using the PCR-SSO technique and chemiluminescence. Exons 2 and 3 of HLA-A and B genes were amplified.

## Results

The HLA-A*02 allele was frequently observed in all groups, being significantly higher among HTLV-1+, and among HTLV-1+ Kollas compared to non-infected ones (p=0.03). HLA-A*31 and *68 were significantly more frequent among negative individuals. HLA-B*07 was higher in HTLV-2+ individuals. HLA-B*35 was higher in HTLV-1 compared to HTLV-2 and in HTLV-1 infected Kollas compared to HTLV-1 infected Caucasians but similar to negative Kollas. HLA-B*40 was higher in negative individuals compared to HTLV-2+ ones.

## Discussion

These data suggest a possible association of HLA-A*02 to susceptibility to infection while HLA-A*31 and *68 may have a possible protective effect against HTLV-1 natural infection. HLA-B*07 might increase HTLV-2 susceptibility to infection. These results show that the allele HLA-B*35 is associated to ethnic groups while the presence of alleles HLA-A*02 and HLA-B*07 may increase susceptibility to HTLV-1 and HTLV-2 infection, respectively. Our observations support that the HLA haplotype modulates the susceptibility to HTLV-1/2 infection.

